# Vibrio cholerae O139 in Calcutta, 1992-1998: incidence, antibiograms, and genotypes.

**DOI:** 10.3201/eid0602.000206

**Published:** 2000

**Authors:** A Basu, P Garg, S Datta, S Chakraborty, T Bhattacharya, A Khan, S Ramamurthy, S K Bhattacharya, S Yamasaki, Y Takeda, G B Nair

**Affiliations:** National Institute of Cholera and Enteric Diseases, Calcutta, India.

## Abstract

We report results of surveillance for cholera caused by Vibrio cholerae O139 from September 1992, when it was first identified, to December 1998. V. cholerae O139 dominated as the causative agent of cholera in Calcutta during 1992-93 and 1996- 97, while the O1 strains dominated during the rest of the period. Dramatic shifts in patterns of resistance to cotrimoxazole, neomycin, and streptomycin were observed. Molecular epidemiologic studies showed clonal diversity among the O139 strains and continuous emergence of new epidemic clones, reflected by changes in the structure, organization, and location of the CTX prophages in the V. cholerae O139


### *Vibrio cholerae* O139 in Calcutta, 1992-1998: Incidence, Antibiograms, and Genotypes

**Arnab Basu,* Pallavi Garg,* Simanti Datta,* Soumen Chakraborty,* Tanuja Bhattacharya,* Asis Khan,* T. Ramamurthy,* S.K. Bhattacharya,* Shinji Yamasaki,† Yoshifumi Takeda,‡ and G. Balakrish Nair***
*National Institute of Cholera and Enteric Diseases, Calcutta, India; †International Medical Center of Japan, Tokyo, Japan; and ‡National Institute of Infectious Diseases, Tokyo, Japan

We report results of surveillance for cholera caused by *Vibrio cholerae* O139 from September 1992, when it was first identified, to December 1998. *V. cholerae* O139 dominated as the causative agent of cholera in Calcutta during 1992-93 and 1996-97, while the O1 strains dominated during the rest of the period. Dramatic shifts in patterns of resistance to cotrimoxazole, neomycin, and streptomycin were observed. Molecular epidemiologic studies showed clonal diversity among the O139 strains and continuous emergence of new epidemic clones, reflected by changes in the structure, organization, and location of the CTX prophages in the *V. cholerae* O139 chromosome.

*Vibrio cholerae*, the gram-negative organism that causes cholera, is well defined on the basis of biochemical tests and DNA homology studies (1), but the serogroups of the species differ in their pathogenic potential. Of the 193 recognized "O" serogroups of *V. cholerae* (2), only O1 and O139 cause epidemic and pandemic cholera. *V. cholerae* O139 was first identified in September 1992 in southern India (3) and rapidly spread to all cholera-endemic areas in India (4) and neighboring countries (5). In February 1994, a new clone of *V. cholerae* O1 El Tor biotype (6) replaced the O139 serogroup as the dominant serogroup causing cholera in Calcutta (7). After a 33-month quiescent period, a new clone of *V. cholerae* O139 (8,9) appeared in August 1996 in Calcutta (10) and was the dominant serogroup until September 1997. This new clone has also spread to other parts of India (11).

The National Institute of Cholera and Enteric Diseases, Calcutta, conducts continuous surveillance for cholera in Calcutta in particular, and in India in general. During September-October 1998, we observed an increase in the incidence of O139 cholera, prompting study of these O139 strains. We report the findings of surveillance performed from September 1992 to December 1998 in India, which enabled us to track and catalog changes in the O139 strains since its identification.

Address for correspondence: G. Balakrish Nair, National Institute of Cholera and Enteric Diseases, P-33, CIT Road, Scheme XM, Beliaghata, Calcutta, 700 010, India; fax: 91-33-350-5066; e-mail: gbnair@vsnl.com.

### The Study

### Hospital Surveillance and Bacteriology

Stool specimens were obtained from patients admitted to the Infectious Diseases Hospital, Calcutta, the only hospital that admits cholera patients from the city and its suburbs. Since 1995, on two randomly selected days per week, every fifth patient has been enrolled from all patients with diarrhea or dysentery, with or without other complaints, visiting the emergency department of the Infectious Diseases Hospital, Calcutta. Before 1995, only patients with diarrhea admitted to the Infectious Diseases Hospital, Calcutta, from Monday to Friday between 9 a.m. and 1 p.m. were enrolled. Methods of collection, transport, and bacteriologic examination of stool samples and identification and serotyping of *V. cholerae* have been described (7). The National

Institute of Cholera and Enteric Diseases, Calcutta, is India's national reference laboratory for cholera, and strains from throughout the country are sent here for confirmation and phage typing. Strains received are characterized by an array of tests (4). In this study, representative strains of *V. cholerae* O139 isolated from hospitalized patients in Calcutta from 1992 to 1998 and 15 *V. cholerae* O139 strains from other parts of India (eastern, central, northern, and southern India) isolated in 1998 were randomly selected for molecular characterization.

### Antimicrobial Susceptibility

The *V. cholerae* O139 strains were examined for resistance to ampicillin (10 μg), chloramphenicol (30 μg), cotrimoxazole (25 μg), ciprofloxacin (5 μg), furazolidone (100 μg), gentamycin (10 μg), neomycin (30 μg), nalidixic acid (30 μg), norfloxacin (10 μg), streptomycin (10 μg), and tetracycline (30 μg) by using antibiotic-impregnated commercial disks (Hi Media, Mumbai, India). Characterization of the strains as susceptible or resistant was based on the size of inhibition zones around each disk, according to the manufacturer's instructions, which followed World Health Organization recommendations (12). Strains showing an intermediate zone of inhibition were interpreted as resistant on the basis of previous minimum inhibitory concentration studies conducted with *V. cholerae* (13).

### Polymerase Chain Reaction (PCR) Assay

PCR assays (both multiple and single) were used to screen the O139 strains for five important virulence genes. The first PCR assay used three pairs of oligonucleotide primers for virulence genes *ctxA* (301 bp), *tcpA* (classical; 617 bp), and *tcpA* (El Tor; 471 bp), and the second and third PCR assays used primer pairs for the virulence genes *zot* and *ace*, respectively (Table 1) (15). The composition of the reaction mix and cycling conditions for amplification have been described (16).

Amplification was done by using an automated thermal cycler (Biometra Gottingen, Germany). *V. cholerae* strain VC20 (El Tor, Ogawa), 569B (classical, Inaba) and *Escherichia coli* DH5-α strains were used as positive and negative controls. Amplified products were electrophoresed in a 2% agarose gel (SRL, Bombay, India) in 1x TAE buffer along with standard molecular weight markers (MWM), stained with ethidium bromide (Sigma), and recorded with a video documentation system (Pharmacia Biotech, San Francisco, CA).

Table 1. Oligonucleotide primer sequences used in polymerase chain reaction assays of *Vibrio cholerae* O139

| Gene | Primer sequence (5'-3') | Amplicon size (bp) | Ref. |
|---|---|---|---|
| *ctx*A | CTCAGACGGGATTTGTTAGGCACG | 301 | 14 |
| | TCTATCTCTGTAGCCCCTATTACG | | |
| *tcp*A (El Tor) | GAAGAAGTTTGTAAAAGAAGAACAC | 471 | 14 |
| | GAAGGACCTTCTTTCACGTTG | | |
| *tcp*A (classical) | CACGATAAGAAAACCGGTCAAGAG | 617 | 16 |
| | ACCAAATGCAACGCCGAATGGAG | | |
| *ace* | GCTTATGATGGACACCCTTTA | 282 | 15,[a] |
| | TTTAACGCTCGCAGGGC | | |
| *zot* | GGGCGAGAAAGGACGC | 834 | [a] |
| | CCTTGTAGCGGTAGCTCG | | |

[a]Referred to in this study.

### Pulsed-Field Gel Electrophoresis (PFGE)

Genomic DNA of various strains of *V. cholerae* was prepared in agarose plugs (17). For complete digestion of the DNA, 50U of *Not*I enzyme was used. PFGE of inserts was done by the contour-clamped homogeneous electric field method on a CHEF-mapper (Bio-Rad) with 0.5x TBE Buffer [44.5 mM Tris, 44.5 mM boric acid, 1 mM EDTA (pH 8.0)] for 40.24 hours. A DNA size standard (λ-ladder; Bio-Rad) was used as molecular weight standard. A model 1,000 minichiller (Bio-Rad) was used to maintain the temperature of buffer at 14°C. Run conditions were generated by the auto-algorithm mode of a CHEF Mapper PFGE system with a size range of 20-300 kb. Gels were stained in distilled water containing 1.0 μg ethidium bromide per ml for 30 minutes, rinsed several times, and photographed. After electrophoresis, ethidium bromide staining and photography, transfer of DNA from gel to Hybond $N^+$ membrane (Amersham International PLC, Buckinghamshire, England) and Southern blot hybridization with an O139-specific probe were done as described (17).

The DNA adjacent to Tn5*lac* insertion in MO10 (an O139 strain from the Madras outbreak) rendered the strain O139 negative in agglutinability with O139-specific antiserum. The O139 probe used in this study was prepared as described earlier (18).

### Restriction Fragment Length Polymorphism (RFLP) of *ctxA* Gene and O139 Specific Gene

A *ctxA* probe consisting of a 540-bp *Xba*I-*Cla*I fragment of ctxA cloned in pKTN901 with *Eco*RI

linkers was used (19). A modification of the method of Murray and Thompson (20) was used for DNA extraction for *ctx* RFLP (16). The transfer of DNA from gel to Hybond N+ membrane (Amersham) and hybridization with probes was done (16) with the ECL Nucleic Acid Detection System (Amersham). The membranes were then washed and exposed to Kodak Biomax film (Eastman Kodak Co., Rochester, NY) and developed according to manufacturer's instructions.

### Results

The O139 serogroup dominated as the major cholera-causing serogroup in 1993 and from September 1996 to September 1997, while the O1 serogroup dominated during the remaining part of the study period (Figures 1, 2). The rate of isolation of O139 serogroup was lower in 1998 than in any other year since 1992, the year it was identified (Table 2). The seasonality in incidence of the O139 serogroup showed an interesting trend: apart from 1993 and 1994, more O139 cholera cases were recorded during the latter half of the year, coinciding with the second peak of cholera cases (Figures 1, 2). However, the peak incidence of cholera caused by the O1 serogroup was generally observed during June-July, except for 1993 and 1997, when the O1 peak occurred during October and September, respectively (Figures 1, 2). The O139 strains were the dominant cholera-causing serogroup during these years (Table 2; Figures 1, 2).

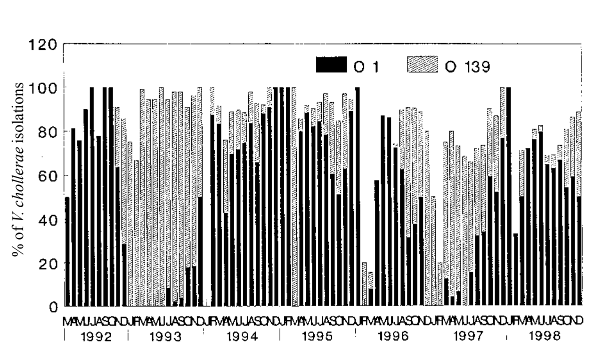

Figure 2. Monthly isolation profile of the percentage of *V. cholerae* O1 and O139 serogroups from patients hospitalized with acute secretory diarrhea at the Infectious Diseases Hospital, Calcutta, India, from March 1992 to December 1998.

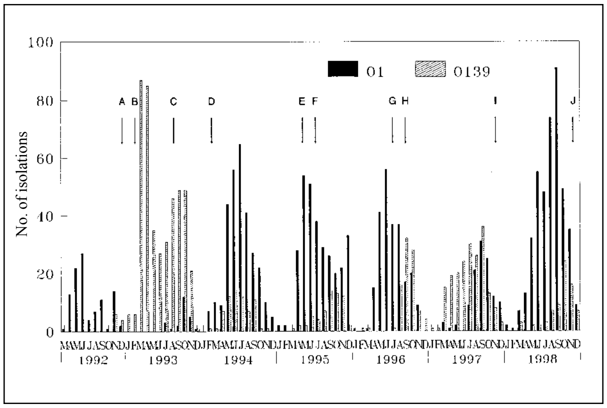

Figure 1. Monthly isolation profile of the *Vibrio cholerae* O1 and O139 serogroups from patients hospitalized with acute secretory diarrhea at the Infectious Diseases Hospital, Calcutta, India, from March 1992 to December 1998. Arrows denote the month in which changes in *V. cholerae* strains were noted: A, appearance of *V. cholerae* O139 in Calcutta in November 1992; B, displacement of *V. cholerae* O1 by *V. cholerae* O139 in January 1993; C, reappearance of *V. cholerae* O1; D, domination of *V. cholerae* O1 over *V. cholerae* O139; E, isolation of nontoxigenic *V. cholerae* O139 in April 1995; F, appearance for the first time of cotrimoxazole-susceptible strains of *V. cholerae* O139; G, appearance of cotrimoxazole-susceptible and neomycin-resistant strains of *V. cholerae* O139; H, domination of *V. cholerae* O139 over *V. cholerae* O1; I, replacement of *V. cholerae* O139 by *V. cholerae* O1 as the major cholera causing serogroup; J, appearance of cotrimoxazole- and streptomycin-susceptible strains of *V. cholerae* O139.

The antibiotic resistance patterns of randomly selected O139 strains isolated from Calcutta during 1992-1998 (Figure 3) showed dramatic changes in resistance to cotrimoxazole, with all the 1992, 1993, and 1994 O139 strains being resistant, while most of the strains isolated during 1997 and 1998 were sensitive. Likewise, resistance to neomycin and streptomycin also increased until 1996, then declined. O139 strains isolated throughout the study were resistant to furazolidone and most were resistant to ampicillin. The dominant drug-resistance patterns among O139 strains isolated in Calcutta from 1993 to 1998 were A C Co Fz S (30%), A C Co Fz S (48.1%), A Co Fz S (49.1%), A Fz N S (96%), A Fz N S (32%), and A Fz (98.3%).

PCR studies with 106 representative O139 strains showed that all but one strain (CO853)

Table 2. Isolation rates of *Vibrio cholerae* O139 from patients with acute secretory diarrhea admitted to the Infectious Diseases Hospital, Calcutta, 1992–1998

| | No. of | No. (%) of samples positive for *V. cholerae* | | | |
|---|---|---|---|---|---|
| | samples | Total | | | |
| Year | screened | positive | O1 | O139 | Non-O1 |
| 1992 | 359 | 131 (36.5) | 102 (28.4) | 10 (2.8) | 19 (5.3) |
| 1993 | 890 | 491 (55.2) | 24 (2.7) | 443 (49.8) | 24 (2.7) |
| 1994 | 748 | 400 (53.5) | 296 (39.7) | 67 (8.9) | 37 (4.9) |
| 1995 | 873 | 399 (45.7) | 305 (34.9) | 62 (7.1) | 32 (3.7) |
| 1996 | 1746 | 399 (22.9) | 234 (13.4) | 62 (3.6) | 103 (5.9) |
| 1997 | 1643 | 397 (24.2) | 114 (6.9) | 196 (11.9) | 87 (5.3) |
| 1998 | 2118 | 642 (30.3) | 416 (19.7) | 73 (3.4) | 153 (7.2) |

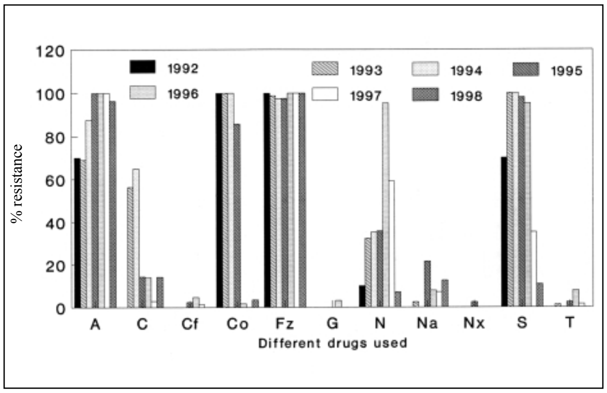

Figure 3. Antibiotic resistance pattern of the *V. cholerae* O139 strains. Abbreviations: A, ampicillin; C, chloramphenicol; Cf, ciprofloxacin; Co, cotrimoxazole; Fz, furazolidone; G, gentamycin; N, neomycin; Na, nalidixic acid; Nx, norfloxacin; S, streptomycin; T, tetracycline.

were positive for the 471-bp *tcpA* (El Tor) amplicon, indicating that almost all have the organelle required for intestinal colonization. The presence of 301-bp *ctxA*, 843-bp *zot*, and 282-bp *ace* amplicons in all strains except CO853 indicates that all the *tcpA*-positive strains have an intact CTX prophage.

The PFGE profile of three randomly selected O139 strains, sharing the unique $CTX^{ImmCalcutta}$ prophage and isolated from Calcutta during 1996 and 1997, has a banding pattern similar to that of the reference strain MO45 (ATCC 51394) isolated during 1992 in Madras (Figure 4A). The band patterns exhibited by all the O139 strains differed from that of the classical and El Tor biotype representative strains of *V. cholerae* O1, 569B, 2164-88, and CO840. However, the pattern of the reference O139 strain MO45 was identical to that of the O1 strain MO1, which was described as the progenitor strain of the O139 serogroup (21). The Southern blot of the PFGE DNA fragments with O139-specific probe showed that the probe hybridized at the same position in all the O139 strains. However, the control O1 strains and the progenitor strain MO1 did not hybridize with the probe (Figure 4B).

RFLP was then conducted to determine any differences in number, location, and arrangement of the CTX prophage in the genome of O139 strains. As the CTX prophage of the 1992-93 and 1996 O139 strains is well characterized (8,11,16,22), we took only one representative strain from 1992 (SG24) and 1996 (AM258) (8,11,22). Five O139 strains (PG265, PG269, PG316, PG337, and PG351) isolated during September 1998 (when an upsurge in O139 strains was observed) were included in the study. RFLP of the CTX prophage with *Hin*dIII showed one band with SG24, PG265, PG269, PG316, and PG351; AM258 and PG337 had three bands of 14, 9.2, and 7.0 kb (Table 3). As *Hin*dIII has no site in the CTX prophage (23), four of the five 1998 O139 strains (PG265, PG269, PG316, and PG351), like the 1992 O139 strain (SG24), have the CTX prophage at only one site in the chromosome. RFLP with *Pst*I and *Bgl*II showed only one band each of 5.6 kb and 8 kb, respectively, in four of the 1998 O139 strains (PG265, PG269, PG316, and PG351), while the 1992 O139 strain (SG24) exhibited two bands of sizes 7.0 and 5.6 and 8.0 and 7.0 kb, respectively (Table 3). As *Pst*I and *Bgl*II have only a single site in the CTX prophage but not within the *ctxA* gene, these results indicate that most of the 1998 O139 strains have only one CTX prophage, unlike the 1992 O139 strain, which has two CTX prophages arranged in tandem (Figure 5). Further, as both the 8- and 5.6-kb bands are shared by the 1992 and 1998 *V. cholerae* O139 strains, the downstream regions of the CTX prophage in the 1992 and 1998 *V. cholerae* O139 strains are 97% similar,

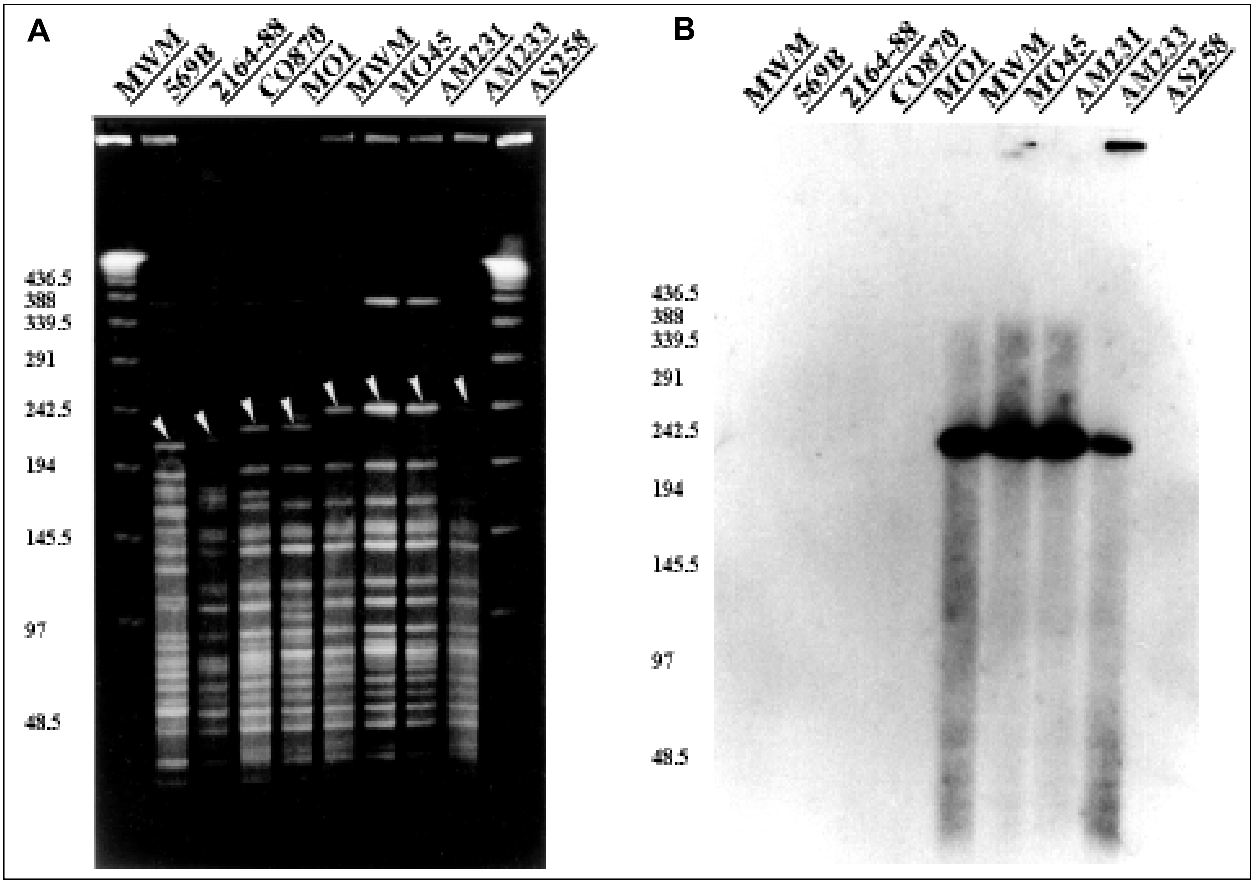

Figure 4. Pulsed-field gel electrophoresis profiles of the *V. cholerae* O139 strains (A) and Southern blot of the same gel with O139-specific probe. (B). The numbers at the top indicate the strain numbers. Lanes 1, MWM (bacteriophage λ ladder); 2, 569B (O1 classical Inaba); 3, 2164-88 (O1 El Tor Ogawa); 4, CO870 (O1 El Tor Ogawa); 5, MO1 (progenitor of the O139 strain); 6, MO45 (O139 strain isolated during 1992-93); 7, AM231 (O139 strain isolated during 1996-97); 8, AM233 (O139 strain isolated during 1996-97); 9, AS258 (O139 strain isolated during 1996-97); 10, MWM (bacteriophage λ ladder).

Table 3. Fragment sizes of restriction enzyme-digested genomic DNA of *Vibrio cholerae* O139 strains, isolated by *ctxA* probe, Calcutta, 1992-1998

| | | | Fragment (kb) hybridized with the probe | | |
|---|---|---|---|---|---|
| Strain | Yr. isolated | Antibiogram | *Hin*dIII | *Bgl*II | *Pst*I |
| SG24 | 1992 | CoFzS | 23 | 8.0, 7.0 | 9.3, 5.6 |
| AM258 | 1996 | ACCfFzNS | 14.0, 9.2, 7.0 | 23.0 | 7.0, 5.6 |
| PG265 | 1998 | AFz | 18.0 | 8.0 | 5.6 |
| PG269 | 1998 | AFz | 18.0 | 8.0 | 5.6 |
| PG316 | 1998 | AFz | 18.0 | 8.0 | 5.6 |
| PG337 | 1998 | AFzNS | 14.0, 9.2, 7.0 | 23 | 7.0, 5.6 |
| PG351 | 1998 | AFz | 16.0 | 8.0 | 5.6 |

suggesting that they may share the identical location in the *V. cholerae* genome. Southern blot of *Hin*dIII-digested genomic DNA, using a *ctxA* probe of the 1996 O139 strain (AM258), showed three bands, and the Southern blots of the *Pst*I- and *Bgl*II-digested genomic DNA, also using the *ctxA* probe, showed only two bands. As discussed (8,16), the strain has the same organization of the CTX prophage as the 1996-97 O139 strains isolated from Calcutta. It has three tandemly duplicated CTX prophages, with the second and third CTX prophages being new and differing from the first in having an altered restriction endonuclease site (8,9) (Figure 5). The Southern blots of the 1998 O139 strain PG337 exactly match those of the 1996-97 representative strain AM258 (Table 3), indicating that its structure resembles that of the 1996-O139 strains.

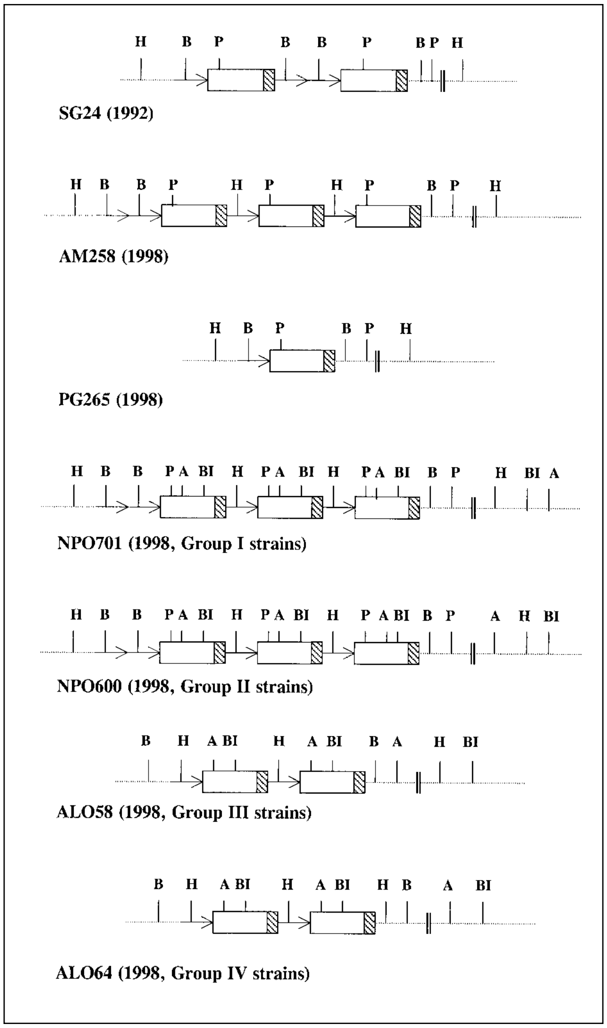

Figure 5. Schematic diagram of CTX prophage (not to scale) as deduced from Southern blot hybridization data. Arrows and boxes correspond to the restriction sites and core regions of CTX prophage, respectively, with hatched portions in the box representing *ctxAB* genes. Restriction site abbreviations: A, *Ava*I; BI, *Bgl*I; B, *Bgl*II; H, *Hin*dIII; P, *Pst*I.

Southern blot analysis of *Hin*dIII-digested genomic DNA of the 15 O139 strains isolated from different parts of India in 1998 showed four variations when probed with *ctxA*. Of the 15 strains examined, eight (designated group I strains) showed three bands of size 14, 9.2, and 7.0 kb (the pattern displayed by the 1996-97 O139 strains); two strains had three fragments of sizes 14, 12, and 7.0 kb (designated group II strains); and four had two fragments of sizes 23 and 7.0 kb (designated group III strains); only one had two bands of 14 and 7.0 kb (designated group IV strains) (Table 4). All group I and group II strains showed a single band of about 23 kb in the blot when digested with *Bgl*II and probed with *ctx*A (Table 4), indicating that in these strains the *Bgl*II site resides outside the CTX prophage, as described (8). Thus, group I and group II strains have the same unique organization of the CTX prophage as that of the 1996-97 O139 strains (8). The group III and group IV strains also showed a single band when digested with *Bgl*II and probed with *ctxA*, indicating that in these strains the *Bgl*II site may also be outside the CTX prophage (Table 4). Representative strains of the four types showed a common band of approximately 7 kb when digested with *Bgl*I, *Ava*I, and *Pst*I and probed with the *ctxA* probe (Table 4). Since all these enzymes have a single restriction site in the CTX prophage but not within the *ctxA* gene (23) and the size of an intact CTX prophage is approximately 7 kb, the 7-kb band in all these enzymes indicates a tandem duplication in all strains. All the group I strains have three CTX prophages in tandem (8,16), with the second and third prophages having a *Hin*dIII site instead of the *Bgl*II site in the RS region of the CTX prophage, like the 1996-97 O139 strains (Figure 5). The group II strains differ from the group I strains by the position of the second band in the blots (Table 4). Thus, the group II strains have exactly the same arrangement of the CTX prophage in their genome as the group I strains; the difference in the size of the second band could be due to the integration of the prophage at a different site in the genome (Figure 5).

The group III and group IV strains showed two bands in *Hin*dIII, *Bgl*I, and *Ava*I blots when probed with *ctxA*. There is no *Hin*dIII restriction site in the CTX prophage of El Tor and classical strains (23), while *Ava*I, *Bgl*II and *Bgl*I have only one site within the CTX prophage (23). In the $CTX^{ImmCalcutta}$ prophage, present in the 1996-97 O139 strains, the positions of the *Hin*dIII and the *Bgl*II enzymes have been interchanged (8,9). The presence of two bands in the blots with a common 7.0 kb in *Hin*dIII, *Ava*I, and *Bgl*I blots with *ctxA* and one band in the *Bgl*II blot (Table 4) with same probe indicates that group III and group IV have two copies of the CTX prophage in tandem, with both the CTX prophages in these strains being the new type of the O139 CTX prophage, $CTX^{ImmCalcutta}$ prophage (Figure 5). Thus, these O139 strains (all isolated in southern India) may derive from the 1996-97 O139 strains but lack the

Table 4. Restriction enzyme-digested fragments of genomic DNA of *Vibrio cholerae* O139 strains isolated with the ctxA probe, India, 1998

| Strain no. | Place (Region) of isolation | Anti-biogram | Fragment size (kb) using *ctxA* probe *Hind*III | *Bgl*II | *Bgl*I | *Ava*I | *Pst*I |
|---|---|---|---|---|---|---|---|
| NPO600 | Nagpur (Central) | ACFzNaS | 14, 12,7 | 23 | 23, 7 | 14, 7 | 9.5, 7 |
| NPO701 | Nagpur (Central) | ACNaS | 14, 9.2, 7 | 23 | 12, 7 | 23, 7 | 7, 5 |
| NPO702 | Nagpur (Central) | AFzNaS | 14, 12, 7 | 23 | 23, 7 | 14, 7 | 9.5, 7 |
| NPO706 | Nagpur (Central) | ACFzNaS | 14, 9.2, 7 | 23 | 12, 7 | 23, 7 | 7, 5 |
| NPO708 | Nagpur (Central) | AFzNaS | 14, 9.2, 7 | 23 | 12, 7 | 23, 7 | 7, 5 |
| NPO709 | Nagpur (Central) | AFzNaS | 14, 9.2, 7 | 23 | 12, 7 | 23, 7 | 7,5 |
| MUO7 | Murshidabad (Eastern) | CFzS | 14, 9.2, 7 | 23 | 12, 7 | N.D. | N.D. |
| LHO359 | Ludhiana (Northern) | AFzNaS | 14, 9.2, 7 | 23 | 12, 7 | N.D. | N.D. |
| LHO469 | Ludhiana (Northern) | AFzNS | 14, 9.2, 7 | 23 | 12, 7 | N.D. | N.D. |
| LHO477 | Ludhiana (Northern) | FzNaS | 14, 9.2, 7 | 23 | 12, 7 | N.D. | N.D. |
| ALO53 | Alleppey (Southern) | FzS | 23, 7 | 23 | 23, 7 | 23, 7 | N.D. |
| ALO58 | Alleppey (Southern) | ACFz | 23, 7 | 23 | 23, 7 | 23, 7 | N.D. |
| ALO59 | Alleppey (Southern) | AFzNaS | 23, 7 | 23 | 23, 7 | 23, 7 | N.D. |
| ALO62 | Alleppey (Southern) | FzS | 23, 7 | 23 | 23, 7 | 23, 7 | N.D. |
| ALO64 | Alleppey (Southern) | ACFzS | 14, 7 | 23 | 23, 7 | 23, 7 | N.D. |

El Tor CTX prophage of the three CTX prophages that they have in tandem. Alternatively, these group III and group IV strains may be a new type of O139 that lacks the El Tor CTX prophage. The only difference between the group III and group IV strains is in the *Hin*dIII blot, which may be due to polymorphism in the *Hin*dIII site (Table 4; Figure 5).

### Discussion

The emergence of *V. cholerae* O139 is a puzzling event in the history of cholera. The sudden appearance of the O139 serogroup in late 1992, its rapid spread through Southeast Asia in 1993 followed by a quiescent period during 1994-95 and its subsequent emergence in 1996 and 1997 are inadequately understood, but characterize the unpredictable nature of the epidemiology of cholera. However, the reemergence of O139 in 1996 indicates that its appearance was not a one-time event and the serogroup has the potential to persist and spread to other continents.

Comparison of the O139 strains isolated during the 7-year study period revealed interesting patterns of antibiotic resistance to various common antibiotics. While the strains remained largely susceptible to ciprofloxacin, tetracycline, and gentamycin, resistance to ampicillin and susceptibility to cotrimoxazole (sulphamethoxazole, trimethoprim), chloramphenicol, and streptomycin increased during the period of the study. Resistance to cotrimoxazole (sulphamethoxazole and trimethoprim) and streptomycin is encoded by a 62-kb self-transmissible chromosomally integrated transposon termed the SXT element, which is not apparently linked to genes encoding O139 antigen (24). The SXT element was present in 1992-93 O139 strains and is probably silent or absent in 1998 O139 strains, which were sensitive to cotrimoxazole and streptomycin. The presence of this novel region in 1996 O139 strains is difficult to predict because these strains are susceptible to cotrimoxazole (sulphamethoxazole and trimethoprim) but resistant to streptomycin. However, this pattern of rapid shift is consistent with recent reports indicating an enhanced mobility in genetic elements, which confers resistance to antibiotics (25) and has also been observed in *V. cholerae* O1 strains (7). A multiple-antibiotic resistance plasmid belonging to incompatibility group C has been associated with drug-resistance plasmid of *V. cholerae* O139 (17). Since the multidrug-resistance plasmid is self-transmissible by conjugation, the incidence of plasmid-carrying strains and hence drug resistance may change, depending on the presence of these plasmids in O139 strains.

Molecular studies show continuous change in the structure and organization of CTX prophage during the study period, along with evolution of a new type of CTX prophage. The 1992-93 strains have two CTX prophages connected by an RS1 element, while the 1996 O139 strains have three CTX prophages arranged in tandem (8,22). Most of the 1998 O139 strains from Calcutta have only one CTX prophage, while those isolated from other parts of India have either the arrangement of the 1996-97 O139 strains (group I and II strains) or have two CTX prophages arranged in tandem (group III and IV strains). However, as reported elsewhere (9), the 1996 O139 strains have two types of CTX prophages, with the first of the three an El Tor type CTX prophage and the second and third CTX prophages being a new type of CTX prophage that differs primarily in the *rstR* gene, the gene that codes for the repressor protein of CTX . In 1998, we observed two new clones of O139 at two epicenters, Calcutta and Alleppey. From the restriction profile data of the 1998 O139 strains, it can be predicted that most of the O139 strains from Calcutta have only the El Tor type CTX prophage and not the unique O139 CTX prophage of the 1996 O139 strains, while the reverse is the case with the south Indian (Alleppey) strains. Therefore, two different clones of O139 are circulating at two locations with different types of CTX prophages, indicating that reassortment in the genome is taking place in the O139 strains. Our study indicates a continuous emergence of new clones of toxigenic *V. cholerae*, possibly through natural selection involving unidentified factors and immunity of the host population (25-28). Another possibility is that the genetic reassortments observed here are random changes in the organism. The strains that gained advantage as a result of this rearrangement may have infected humans and become enriched inside the gastrointestinal tract so that they became detectable as new toxigenic strains. Molecular analysis of *V. cholerae* strains isolated during epidemics from 1961 to 1996 in Bangladesh revealed similar clonal diversity among strains isolated during different epidemics (25-29). These studies demonstrated three different ribotypes among the *V. cholerae* O139 isolated from Bangladesh, with different ribotypes often showing different CTX prophage genotypes (26,28).

This work was supported in part by the Japan International Cooperation Agency (JICA/NICED Project 054-1061-E-0).

Mr. Basu holds a Masters in Biochemistry and is completing his doctoral degree in Microbiology under the supervision of G. Balakrish Nair, Deputy Director of the National Institute of Cholera and Enteric Diseases, the National Reference Centre for Cholera in India. Mr. Basu's doctoral thesis is an in-depth molecular analysis of the CTX genetic element in strains of *Vibrio cholerae* isolated in the last 3 decades, including recent isolates of *V. cholerae* O139.

### References

1. Baumann P, Furniss AL, Lee JV. Bergey's manual of systematic bacteriology. Vol 1. In: Genus 1, *Vibrio* P. Klieg NR, Holt JG, editors. Baltimore: Williams and Wilkins; 1984. p. 518-38.
2. Yamai S, Okitsu T, Shimada T, Katsube Y. Distribution of serogroups of *Vibrio cholerae* non-O1 non-O139 with specific reference to their ability to produce cholera toxin and addition of novel serogroups. Journal of Japanese Association of Infectious Diseases 1997;71:1037-45.
3. Ramamurthy T, Garg S, Sharma R, Bhattacharya SK, Nair GB, Shimada T, et al. Emergence of a novel strain of *Vibrio cholerae* with epidemic potential in southern and eastern India. Lancet 1993;341:703-4.
4. Nair GB, Ramamurthy T, Bhattacharya SK, Mukhopadhyay AK, Garg S, Bhattacharya MK, et al. Spread of *Vibrio cholerae* O139 Bengal in India. J Infect Dis 1994;169:1029-34.
5. Nair GB, Albert MJ, Shimada T, Takeda Y. *Vibrio cholerae* O139 Bengal: the new serogroup causing cholera. Medical Microbiolical Reviews 1996;7:43-51.
6. Sharma C, Nair GB, Mukhopadhyay AK, Bhattacharya SK, Ghosh RK, Ghosh A. Molecular characterization of *V. cholerae* O1 biotype El Tor strains isolated between 1992 and 1995 in Calcutta, India: evidence for the emergence of a new clone of the El Tor biotype. J Infect Dis 1997;175:1134-41.
7. Mukhopadhyay AK, Garg S, Mitra R, Basu A, Dutta D, Bhattacharya SK, et al. Temporal shifts in traits of *Vibrio cholerae* strains isolated from hospitalized patients in Calcutta: a 3-year (1993-1995) analysis. J Clin Microbiol 1996;34:2537-43.
8. Sharma C, Maiti S, Mukhopadhyay AK, Basu A, Basu I, Nair GB, et al. Unique organization of the CTX genetic element in *Vibrio cholerae* O139 strains which reemerged in Calcutta, India, in September, 1996. J Clin Microbiol 1997;35:3348-50.
9. Kimsey H, Nair GB, Ghosh A, Waldor MK. Diverse CTXphis and evolution of new pathogenic *Vibrio cholerae*. Lancet 1998;352:457-8.
10. Mitra R, Basu A, Dutta D, Nair GB, Takeda Y. Resurgence of *Vibrio cholerae* O139 Bengal with altered antibiogram in Calcutta, India. Lancet 1996;348:1181.
11. Mukhopadhyay AK, Basu A, Garg P, Bag PK, Ghosh A, Bhattacharya SK, et al. Molecular epidemiology of reemergent *Vibrio cholerae* O139 Bengal in India. J Clin Microbiol 1998;36:2149-52.
12. World Health Organization. Guidelines for cholera control. Geneva: The Organization; 1993.
13. Yamamoto T, Nair GB, Parodi CC, Takeda Y. In vitro susceptibilities to antimicrobial agents of *V. cholerae* O1 and O139. Antimicrob Agents Chemother 1993;39:241-4.
14. Keasler SP, Hall RH. Detecting and biotyping *V. cholerae* O1 with multiplex polymerase chain reaction. Lancet 1993;341:1661.
15. Colombo MM, Mastrandrea S, Santona A, de Amdrade AP, Uzzau S, Rabino S. et al. Distribution of the *ace, zot*, and *ctxA* toxin genes in the clinical and environmental *Vibrio cholerae*. J Infect Dis 1994;170:750-1.

16. Basu A, Mukhopadhyay AK, Sharma C, Jyot J, Gupta N, Ghosh A, et al. Heterogeneity in the organization of the CTX genetic element in strains of *Vibrio cholerae* O139 Bengal isolated from Calcutta, India and Dhaka, Bangladesh and its plausible link to the dissimilar incidence of O139 cholera in the two locales. Microb Pathog 1998;24:175-83.
17. Yamasaki S, Nair GB, Bhattacharya SK, Yamamoto S, Kurazono H, Takeda Y. Cryptic appearance of a new clone of *Vibrio cholerae* O1 biotype El Tor in Calcutta, India. Microbiol Immunol 1997;41:1-6.
18. Waldor MK, Mekalanos JJ. *Vibrio cholerae* O139 specific gene sequence. Lancet 1994;343:1366.
19. Kaper JB, Morris JG Jr, and Nishibuchi M. DNA probes for pathogenic *Vibrio* species. In: Tenover FC, editor. DNA probes for infectious disease. Boca Raton (FL): CRC Press, Inc.; 1992. p. 65-77.
20. Murray MG, Thompson WF. Rapid isolation of high molecular weight plant DNA. Nucleic Acids Res 1980;8:4321-5.
21. Pajni S, Sharma C, Bhasin N, Ghosh A, Ramamurthy T, Nair GB, et al. Studies on the genesis of *Vibrio cholerae* O139: identification of probable progenitor strains. J Med Microbiol 1994;42:20-5.
22. Bhadra RK, Roychoudhury S, Banerjee RK, Kar S, Majumdar R, Sengupta S, et al. Cholera toxin (CTX) genetic element in *Vibrio cholerae* O139. Microbiology 1995;141:1977-83.
23. Mekalanos JJ. Duplication and amplification of cholera toxin genes in *Vibrio cholerae*. Cell 1983;35:253-63.
24. Waldor MK, Tschape H, Mekalanos JJ. A new type of conjugative transposon encodes resistance to sulfamethoxazole, trimethoprim and streptomycin in *Vibrio cholerae* O139. J Bacteriol 1996;178:4157-65.
25. Faruque SM, Alim ARMA, Rahman MM, Siddique AK, Sack RB, Albert MJ. Clonal relationships assay classical *Vibrio cholerae* O1 strains isolated between 1961 and 1992 in Bangladesh. J Clin Microbiol 1993;31:2513-6.
26. Faruque SM, Ahmed KM, Siddique AK, Zoman K, Alim ARMA, Albert MJ. Molecular analysis of toxigenic *Vibrio cholerae* in Bangladesh studied by numerical analysis of rRNA gene restriction patterns. J Clin Microbiol 1995;33:2833-8.
27. Faruque SM, Roy SK, Alim ARMA, Siddique AK, Albert MJ. Molecular epidemiology of toxigenic *Vibrio cholerae* in Bangladesh studied by numerical analysis of rRNA gene restriction patterns. J Clin Microbiol 1995;33:2833-8.
28. Faruque SM, Ahmed KM, Alim ARMA, Qadri F, Siddique AK, Albert MJ. Emergence of a new clone of toxigenic *Vibrio cholerae* O1 biotype El Tor displacing *V. cholerae* O139 Bengal in Bangladesh. J Clin Microbiol 1997;35:624-30.
29. Faruque SM, Alim ARMA, Roy SK, Khan F, Nair GB, Sack RB, et al. Molecular analysis of rRNA and cholera toxin genes carried by the new epidemic strain of toxigenic *Vibrio cholerae* O139 synonym Bengal. J Clin Microbiol 1994;33:1050-3.
